# Functional Connectome of the Human Brain with Total Correlation

**DOI:** 10.3390/e24121725

**Published:** 2022-11-25

**Authors:** Qiang Li, Greg Ver Steeg, Shujian Yu, Jesus Malo

**Affiliations:** 1 Image Processing Laboratory, University of Valencia, 46980 Valencia, Spain; 2 Information Sciences Institute, University of Southern California, Marina del Rey, CA 90292, USA; 3 Machine Learning Group, UiT—The Arctic University of Norway, 9037 Tromsø, Norway

**Keywords:** Total Correlation, CorEx, fMRI, functional connectivity, large-scale connectome, biomarkers

## Abstract

Recent studies proposed the use of Total Correlation to describe functional connectivity among brain regions as a multivariate alternative to conventional pairwise measures such as correlation or mutual information. In this work, we build on this idea to infer a large-scale (whole-brain) connectivity network based on Total Correlation and show the possibility of using this kind of network as biomarkers of brain alterations. In particular, this work uses Correlation Explanation (CorEx) to estimate Total Correlation. First, we prove that CorEx estimates of Total Correlation and clustering results are trustable compared to ground truth values. Second, the inferred large-scale connectivity network extracted from the more extensive open fMRI datasets is consistent with existing neuroscience studies, but, interestingly, can estimate additional relations beyond pairwise regions. And finally, we show how the connectivity graphs based on Total Correlation can also be an effective tool to aid in the discovery of brain diseases.

## 1. Introduction

The human brain is a complex system comprised of interconnected functional units. Millions of neurons in the brain interact with each other at both a structural and functional level to drive efficient inference and processing in the brain. Furthermore, the functional connectivity among these regions also reveals how they interact with each other in specific cognitive tasks. Functional connectivity refers to the statistical dependency of activation patterns between various brain regions that emerges as a result of direct and indirect interactions [1,2]. It is usually measured by how similar neural time series are to each other, and it shows how the time series statistically interact with each other.

A variety of ways to analyze functional connectivity exist. A seedwise analysis can be performed by selecting a seed-driven hypothesis and analyzing its statistical dependencies with all other voxels outside its limits. It is a common tool for studying how different parts of the brain are connected to one another. Connectivity is determined by calculating the correlation between the time series of each voxel in the brain and the time series of a single seed voxel. Another option is to perform a wide analysis of the voxel or Region Of Interest (ROI), where statistical dependencies on all voxels or ROIs are studied [3]. Structural connectivity refers to the anatomical organization of the brain by means of fiber tracts [4]. The sharing of communication between neurons in multiple regions is coordinated dynamically via changes in neural oscillation synchronizations [5]. When it comes to the brain connectome, functional connectivity refers to how different areas of the brain communicate with one another during task-related or resting-state activities [6]. The use of information-theoretic metrics can efficiently detect their interaction in dynamical brain networks, and it is widely used in the field of neuroscience [7], for instance to quantify information encoding and decoding in the neural system [8,9,10,11], measure visual information flow in the biological neural networks [12,13] and color information processing in the neural cortex [14], and so on. However, although functional connectivity has already become a hot research topic in neuroscience [15,16], systematic studies on the information flow or the redundancy and synergy amongst brain regions remain limited. One extreme type of redundancy is full synchronization, where the state of one neural signal may be used to predict the status of any other neural signal, and this concept of redundancy is thus viewed as an extension of the standard notion of correlation to more than two variables [17]. Synergy, on the other hand, is analogous to those statistical correlations that govern the whole, but not its constituent components [18]. High-order brain functions are assumed to require synergies, which give simultaneous local independence and global cohesion, but are less suitable for them under high synchronization situations, such as epileptic seizures [19]. Most functional connectivity approaches until now have mainly concentrated on pairwise relationships between two regions. The conventional approach used to estimate indirect functional connectivity among brain regions is the Pearson Correlation (CC) [20] and Mutual Information (I) [8,21,22,23]. However, real brain network relationships are often complex, involving more than two regions, and the pairwise dependencies measured by correlation or mutual information cannot reflect these multivariate dependencies. Therefore, recent studies in neuroscience focus on the development of information-theoretic measures that can handle more than two regions simultaneously such as Total Correlation [24,25].

Total Correlation (TC) [26] (also known as multi-information [27,28,29]) mainly describes the amount of dependence observed in the data and, by definition, can be applied to multiple multivariate variables. Its use to describe functional connectivity in the brain was first proposed as an empirical measure in [24], but in [25], the superiority of TC over mutual information was proven analytically. The consideration of low-level vision models allows deriving analytical expressions for TC as a function of the connectivity. These analytical results show that pairwise I cannot capture the effect of different intra-cortical inhibitory connections, while TC can. Similarly, in analytical models with feedback, synergy can be shown using TC, while it is not so obvious using mutual information [25]. Moreover, these analytical results allow calibrating computational estimators of TC.

In this work, we build on these empirical and theoretical results [24,25] to infer a larger-scale (whole-brain) network based on TC for the first time. As opposed to [24,25], where the number of considered nodes was limited to the range of tens and focused on specialized subsystems, here, we consider wider recordings [30,31], so we use signals coming from hundreds of nodes across the whole brain. Additionally, we apply our analysis to data of the same scale for regular and altered brains (http://fcon_1000.projects.nitrc.org/indi/ACPI/html/ accessed on 12 March 2021). We also show the possibility of using this kind of wide-range networks as biomarkers. From the technical point of view, here, we use Correlation Explanation (CorEx) [32,33] to estimate TC in these high-dimensional scenarios. Furthermore, graph theory and clustering [15,16] are used here to represent the relationships between the considered regions.

The rest of this paper is organized as follows: Section 2 introduces the necessary information-theoretic concepts and explains CorEx. Section 3 and Section 4 show two synthetic experiments that prove that the CorEx results are trustable. Section 5 estimates the large-scale connectomes with fMRI datasets that involve more than 100 regions across the whole brain. Moreover, we show how the analysis of these large-scale networks based on TC may indicate brain alterations. Section 6 and Section 7 give a general discussion and the conclusion of the paper, respectively.

## 2. Total Correlation as Neural Connectivity Descriptor

### 2.1. Definitions and Preliminaries

**Mutual Information**: Given two multivariate random variables $X_{1}$ and $X_{2}$, the mutual information between them, $I(X_{1};X_{2})$, can be calculated as the difference between the sum of individual entropies, $H(X_{i})$, and the entropy of the variables considered jointly as a single system, $H(X_{1},X_{2})$ [34]:

(1) $$I\left(X_{1};X_{2}\right)=H\left(X_{1}\right)+H\left(X_{2}\right)-H\left(X_{1},X_{2}\right)$$

where, for each (multivariate) random variable v, the entropy is $H\left(v\right)=\langle -\log_{2}p\left(v\right)\rangle$ and the brackets represent expectation values spanning random variables. The mutual information also can be seen as the information shared by the two variables or the reduction of uncertainty in one variable given the information about the other [35].

**Mutual information is better than linear correlation**: For Gaussian sources, mutual information reduces to linear correlation because the entropy factors in Equation (1) just depend on $\left|\langle X_{1}\cdot X_{2}^{⊤}\rangle \right|$. However, for more general (non-Gaussian) sources, mutual information cannot be reduced to covariance and cross-covariance matrices. In these (more realistic) situations, I is better than the linear correlation because I captures nonlinear relations that are ruled out by $\left|\langle X_{1}\cdot X_{2}^{⊤}\rangle \right|$. For an illustration of the qualitative differences between I and linear correlation, see the examples in Section 2.2 of [24].

As a result, mutual information has been proposed as a good alternative to linear correlation for estimating functional connectivity [8,21]. However, mutual information cannot capture dependencies beyond pairs of nodes. This may be a limitation in complex networks [36].

**Figure 1:**
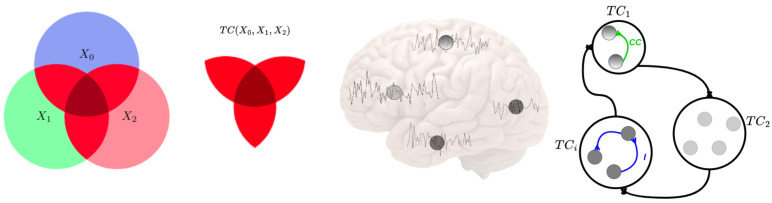
Conceptual scheme of information-theoretic measures of neural information flow. The left circle areas represent the amounts of information, and intersections represent shared information among the corresponding variables, $X_{0},X_{1},X_{2}$. Examples of entropy, $H\left(X_{0}\right),H\left(X_{1}\right),H\left(X_{2}\right)$, Total Correlation (red color), and $TC[X_{0},X_{1},X_{2}]$ are given. The middle figures show some neural time series extracted from brain regions, which correspond to the nodes in the right figure. The right figures illustrate large-scale time series in the brain and how the coupled information is transmitted among the brain regions. The blue and green lines show Linear Correlation (CC) and Mutual Information (I), respectively, between different parts of the brain. The modules represent the lobes of the human brain. Each module has specific brain regions, and each module works with the others.

**Total Correlation**: This magnitude describes the dependence among *n* variables, and it is a generalization of the mutual information concept from two parties to *n* parties. The Venn diagram in Figure 1 qualitatively illustrates this for three variables. The definition of Total Correlation from Watanabe [26] can be denoted as:

(2) $$TC\left(X_{1},\ldots ,X_{n}\right)\equiv \sum_{i=1}^{n}H\left(X_{i}\right)-H\left(X_{1},\ldots ,X_{n}\right)=D_{\mathrm{KL}}\left(p\left(X_{1},\ldots ,X_{n}\right)∥\prod_{i=1}^{n}p\left(X_{i}\right)\right)$$

where $X\equiv \left(X_{1},\ldots ,X_{n}\right)$ and TC can also be expressed as the Kullback–Leibler divergence, $D_{KL}$, between the joint probability density and the product of the marginal densities. From these definitions, if all variables are independent, then TC will be zero.

For the conditional Total Correlation, which is similar to the definition of Total Correlation, but with a condition aed to each term, the Kullback–Leibler divergence of the two conditional probability distributions can also be used to define the conditional Total Correlation. The estimation method used in this work (CorEx presented in the next subsection) uses TC after conditioning on some other variable *Y*, which can be defined as [34]:

(3) $$TC\left(X|Y\right)=\sum_{i}H\left(X_{i}|Y\right)-H\left(X|Y\right)=D_{KL}\left(p\left(x|y\right)∥\prod_{i=1}^{n}p\left(x_{i}|y\right)\right)$$

**Total Correlation is better than mutual information**: This superiority is not only due to the obvious *n*-wise versus pairwise definitions in Equations (1) and (2). It also has to do with the different properties of these magnitudes. To illustrate this point, let us recall one of the analytical examples in [25]. Consider the following feedforward network:

(4) $$X_{1}⟶X_{2}⟶e\overset{f}{⟶}X_{3}$$

where the nodes $X_{1}$, $X_{2}$, *e*, and $X_{3}$ can have any number of neurons, the first two transforms, $X_{1}$⟶$X_{2}$⟶e, are linear and affected by additive noise, and the last transform, f(·), is nonlinear, but deterministic. Imagine that, in this network, one is interested in the connectivity between the neurons in the hidden layer, e; however, the nonlinear function f(·) is unknown, and one only has experimental access to the signal in the regions $X_{1}$, $X_{2}$, and $X_{3}$. In this situation, one could think of measuring $I\left(X_{1},X_{3}\right)=I\left(X_{1},f\left(e\right)\right)$ or $I\left(X_{2},X_{3}\right)=I\left(X_{1},f\left(e\right)\right)$. However, the invariance of *I* under arbitrary nonlinear re-parametrization of the variables [35] implies that these measures are insensitive to *f* and the connectivity therein. On the contrary, as pointed out in [25], using the expression for the variation of TC under nonlinear transforms [13,37], the variation of *H* under nonlinear transforms [34], and the definition in Equation (2), one obtains $TC\left(X_{1},X_{2},X_{3}\right)=\left[TC\left(X_{1},X_{2},e\right)-TC\left(e\right)\right]+TC\left(X_{3}\right)$, where the term in the bracket does not depend on f(·), but the last term definitely does, which proves the superiority of TC over *I* in describing connectivity.

In [25], the network in Equation (4) specifically refers to the flow from the retina, $X_{1}$, to the LGN, $X_{2}$, and finally, to the visual cortex, e and $X_{3}$. However, the result of the superiority of TC over *I* to describe the connectivity in the hidden layer is totally general for every network with the generic properties listed after Equation (4).

### 2.2. Total Correlation Estimated from CorEx

Straightforward application of the direct definition of TC is not feasible in high-dimensional scenarios, and alternatives are required [28,29]. A practical approach to estimate Total Correlation is via *latent factor modeling*. A latent factor model is a statistical model that relates a set of observable variables to a set of latent variables. The idea is to explicitly construct latent factors, *Y*, that somehow capture the dependencies in the data. If we measure dependencies via Total Correlation, TC(X), then we say that the latent factors *explain* the dependencies if TC(X|Y)=0. We can measure the extent to which *Y* explains the correlations in *X* by looking at how much Total Correlation is reduced:

(5) $$\begin{array}{c} TC\left(X\right)-TC\left(X|Y\right)=\sum_{i=1}^{n}I\left(X_{i};Y\right)-I\left(X;Y\right) \end{array}$$

Total Correlation is always non-negative, and the decomposition on the right in terms of mutual information can be verified directly from the definitions. Any latent factor model can be used to lower-bound Total Correlation, and the terms on the right-hand side of Equation (5) can be further lower-bounded with tractable estimators using variational methods; Variational Autoencoders (VAEs) are a popular example [38].

Although latent factor models do not give a direct Total Correlation estimation as the Rotation-based Iterative Gaussianization (RBIG) [28,29] and the matrix-based Rényi entropy [39] did, the approach can be complementary because the construction of latent factors can help in dealing with the curse of dimensionality and for interpreting the dependencies in the data. Compared to CorEx, the main goal of (RBIG https://isp.uv.es/RBIG4IT.htm (accessed on 12 October 2022)) is to convert any non-Gaussian-distributed data into a Gaussian distribution through marginal Gaussianization and rotation to obtain TC. The matrix-based Rényi entropy (http://www.cnel.ufl.edu/people/people.php?name=shujian (accessed on 12 October 2022)) is mainly used for estimating multivariate information based on Shannon’s entropy, which is Rényi’s α-order entropy [40]. With these goals in mind, we now describe a particular latent factor approach known as Total Correlation Explanation (CorEx (https://github.com/gregversteeg/CorEx) (accessed on 12 October 2022)) [32].

CorEx constructs a factor model by reconstructing latent factors using a factorized probabilistic function of the input data, $p\left(y|x\right)=\prod_{j=1}^{m}p\left(y_{j}|x\right)$, with *m* discrete latent factors, $Y_{j}$. This function is optimized to give the tightest lower bound possible for Equation (5).

(6) $$\begin{array}{c} TC\left(X\right)\geq \max_{p(Y_{j}|x)}\sum_{i=1}^{n}I\left(X_{i};Y\right)-I\left(X;Y\right)=\sum_{j=1}^{m}\left(\sum_{i=1}^{n}\alpha_{i,j}I\left(X_{i};Y_{j}\right)-I\left(Y_{j};X\right)\right) \end{array}$$

The factorization of the latent factors leads to the terms $I\left(X;Y\right)=\sum_{j}I\left(Y_{j};X\right)$, which can be directly calculated. The term $I(X_{i};Y)$ is still intractable and is decomposed using the chain rule into $I\left(X_{i};Y\right)\approx \sum \alpha_{i,j}I\left(X_{i};Y_{j}\right)$. Each $I(X_{i};Y_{j})$ can then be tractably estimated [32,33]. There are free parameters $\alpha_{i,j}$ that must be updated while searching for latent factors and achieving objective functions. When t=0, the $\alpha_{i,j}$ initializes and then updates according to:

(7) $$\alpha_{i,j}^{t+1}=\left(1-\lambda \right)\alpha_{i,j}^{t}+\lambda \alpha_{i,j}^{**}$$

The second term $\alpha_{i,j}^{**}=\exp\left(\gamma \left(I\left(X_{i}:Y_{j}\right)-\max_{j}I\left(X_{i}:Y_{j}\right)\right)\right)$, and λ and γ are constant parameters. This decomposition allows us to quantify the contribution to the Total Correlation bound from each latent factor, which can aid interpretability.

CorEx can be further extended into a hierarchy of latent factors [33], helping to reveal the hierarchical structure that we expect to play an important role in the brain. The latent factors at layer *k* explain the dependence of the variables in the layer below.

(8) $$TC\left(X\right)\geq \sum_{k=1}^{r}\left(\sum_{j=1}^{m}\left(\sum_{i=1}^{n}\alpha_{i,j}^{k}I\left(Y_{i}^{k-1};Y_{j}^{k}\right)-\sum_{j=1}^{m}I\left(Y_{j}^{k};Y^{k-1}\right)\right)\right)$$

**Figure 2:**
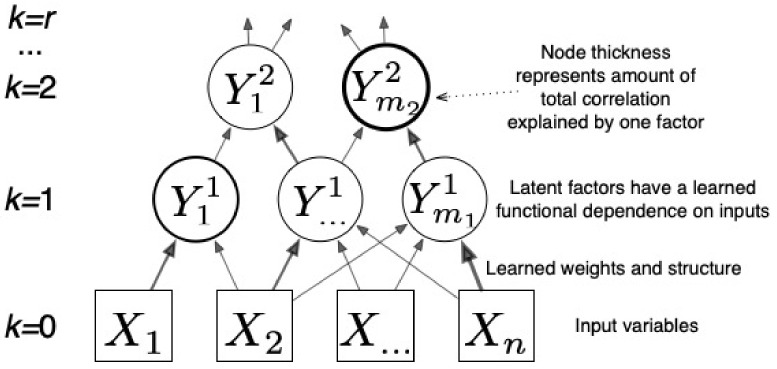
CorEx learns a hierarchical latent factor as illustrated above. Edge thickness indicates strength of the relationship between factors, and node thickness indicates how much Total Correlation is explained by each latent factor.

Here, *k* gives the layer and $Y^{0}\equiv X$ denotes the observed variables. Ultimately, we have a bound on TC that becomes tighter as we add more latent factors and layers and for which we can quantify the contribution for each factor to the bound. We exploit this decomposition for interpretability [41], as illustrated in Figure 2. CorEx prefers to find modular or tree-like latent factor models, which are beneficial for dealing with the curse of dimensionality [42]. For neuroimaging, we expect this modular decomposition to be effective because functional specialization in the brain is often associated with spatially localized regions. We explore this hypothesis in the experiments.

## 3. Experiment 1: Total Correlation for Independent Mixtures

**Figure 3:**
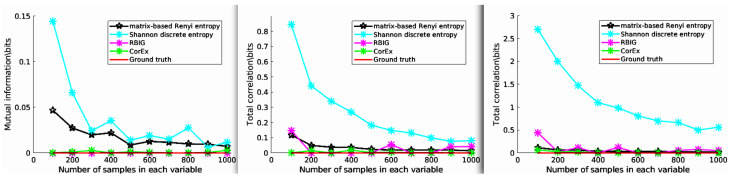
The estimated Total Correlation values for three independent variables. The various Total Correlation estimators are compared with the ground truth value (red line), for example matrix-based Rényi entropy (black line), Shannon discrete entropy (cyan line), RBIG (magenta line), and CorEx (green line). See the main text for more information.

**Figure 4:**
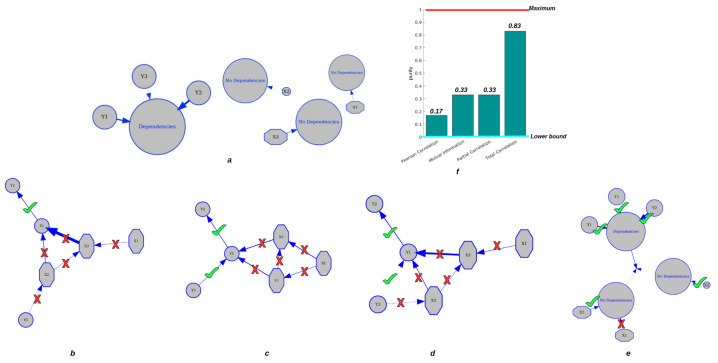
Clustering performance for dependent and independent mixtures. The top row: (**a**) displays the ground truth of variable clustering in two groups. (**f**) shows the purity value of each approach. The second row: (**b**) shows the clustering result based on Pearson correlation. (**c**) shows the clustering result by pairwise mutual information. (**d**) shows the clustering result by partial correlation. (**e**) shows clustering results by CorEx based on Total Correlation.

In this experiment, we estimated the Total Correlation of three independent variables *X*, *Y*, and *Z*, and each follows a Gaussian distribution. For this setup, the ground truth of TC should satisfy TC(X,Y,Z)=0, and we generated various samples with different lengths. Then, the estimated Total Correlation values are shown in Figure 3. Here, we compared CorEx with other different Total Correlation estimators, such as RBIG [28,29], matrix-based Rényi entropy [39], Shannon discrete entropy (https://github.com/nmtimme/Neuroscience-Information-Theory-Toolbox accessed on 12 October 2022), and the ground truth. The left figure (2-dimensional) is mutual information, and the middle (3-dimensional) and right figure (4-dimensional) are Total Correlation. As we mentioned above, the simulation data are totally Gaussian-distributed. Therefore, their dependency should be zero. We find that CorEx and RBIG both perform very well and are very stable, and matrix-based Rényi entropy’s performance becomes more and more nice with increased dimensions, while Shannon discrete entropy becomes more and more accurate with an increase of the samples. All these make sense, and it also explains the accuracy of Total Correlation estimation with CorEx. Here, compared to other estimators, the main functionality goal of CorEx is to cluster statistical dependency variables based on Total Correlation. However, other estimators mainly focus on directly obtaining the Total Correlation value and do not supply very nice visualization results. The CorEx gives us a nice connection with graph theory to visualize and show their functional relationship.

## 4. Experiment 2: Clustering by Total Correlation for Dependent and Independent Mixtures

To evaluate the performance of CorEx in clustering tasks. The elements in group *X* include X1, X2, and X3, which satisfy Gaussian distributions and are completely independent of each other and of group *Y*, and the variables in group *Y* include Y1, Y2 from Y1, and Y3 from Y2, which are connected to each other. Then, we compared the CorEx cluster results with the pairwise Pearson correlation, pairwise mutual information, and partial correlation, which consider confounding effects to find the groups.

In Figure 4, we find that CorEx based on Total Correlation has high accuracy in estimating their dependencies (Figure 4e) compared to pairwise Pearson correlation (Figure 4b), pairwise mutual information (Figure 4c), and partial correlation (Figure 4d). As we established in this experiment, the elements in group *Y* should be clustered together, and the elements in group *X* should be completely independent of each other and of group *Y*. The ground truth is presented in Figure 4a. Then, we estimated the cluster result with the pairwise Pearson correlation with a threshold of 0.1, pairwise mutual information with a threshold of 0.4, and partial correlation without a threshold. Obviously, we found that pairwise approaches have high errors in accurately estimating their statistical dependencies, and pairwise mutual information is better than pairwise Pearson correlation, but still has high errors in correctly clustering tasks. When we considered the confounding effect of the third variables, we still did not obtain a better clustering result compared to TC. Therefore, the clustering results with CorEx by Total Correlation obtain the best performance compared to pairwise approaches. Moreover, we used *purity* as a criterion of clustering quality to qualify the performance of clustering because it is a straightforward and transparent evaluation metric [43]. To calculate purity, each cluster is allocated to the class that occurs most frequently within it, and the accuracy of this assignment is determined by counting the number of correctly assigned elements and dividing by N(N=6). Formally:

(9) $$\mathrm{Purity}\left(X,Y\right)=\frac{1}{N}\sum_{i}\max_{j}\left|X_{i}\cap Y_{j}\right|$$

where X={X1,X2,X3} is the set of clusters and Y={Y1,Y2,Y3} is the set of classes. Figure 4f presents the clustering performance of pairwise approaches and CorEx with purity as a criterion. Poor clusters have near-zero purity ratings (lower bound). A perfect cluster possesses a purity of one (maximum value). Based on Equation (9), we obtain purity values of 0.17 and 0.33 for pairwise approaches and partial correlation, and the purity value for CorEx is 0.83. All in all, we show that CorEx based on Total Correlation has the best performance compared to pairwise approaches.

## 5. Experiment 3: Brain Functional Connectivity Analysis Using Total Correlation

**Figure 5:**
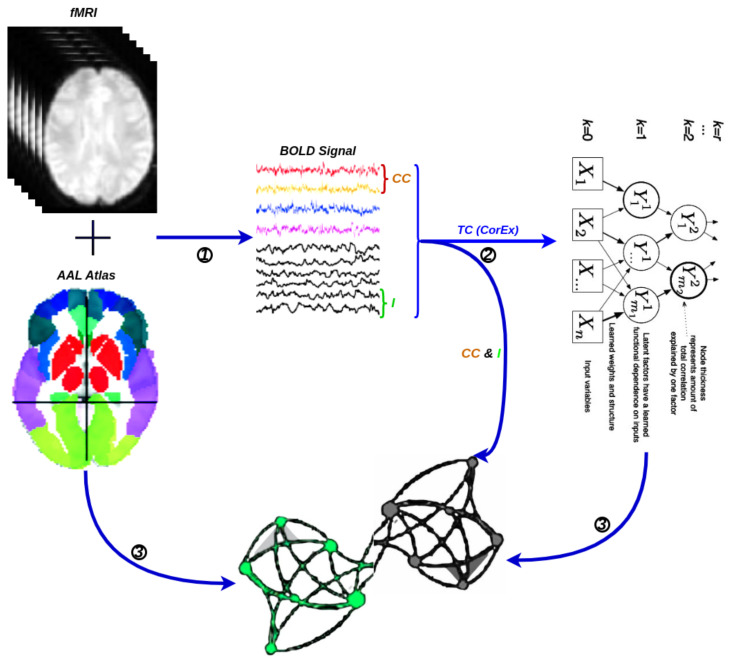
A flowchart for the construction of a functional brain network by fMRI. (**1**) Time series extraction from fMRI data within each anatomical unit (i.e., network node). (**2**) Estimation of functional connectivity with CC, I, and TC (CorEx), respectively. (**3**) Visualization of functional connectivity as tree and circle graphs (i.e., network edges and network nodes).

A network is a collection of nodes and edges, where nodes represent fundamental elements (e.g., brain regions) within the system of interest (e.g., the brain) and edges represent the dependencies that exist between those fundamental elements with the considered weights. Typically, the threshold is chosen based on the visual effect on functional connectivity, and here, we set the optimal threshold for community detection in brain connectivity networks. We used it to identify a threshold that maximizes information on the network modular structure, removes the weakest edges, and keeps the largest connected component. Figure 5 illustrates the schematic representation of network construction using fMRI. Firstly, the time series were extracted from fMRI data based on a selected structural atlas, and then, functional connectivity was estimated with CC, I, and CorEx, respectively. The results are presented with a graph that includes both brain nodes and their functional connectivity with weight edges.

### 5.1. First Total-Correlation-Based Clustering Example from fMRI Data

**Figure 6:**
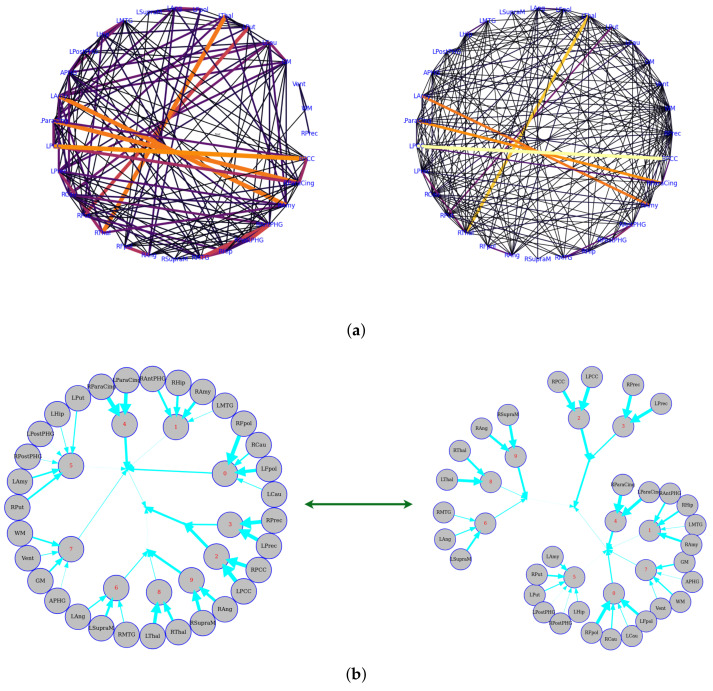
Functional connectivity representation with graph-based networks. The functional connectivity is represented in the cycle (**a**) and tree (**b**) graphs. Top row: the left and right figures correspond to Pearson correlation with a threshold of 0.14 and mutual information with a threshold of 0.02, respectively. Bottom row: the figures show the Total Correlation with a threshold of 0.16 that was estimated by CorEx. To more directly display the statistical dependencies of brain regions, we here converted the circle graph to a tree graph. The weights are shown by the thickness of the edges, which shows how strongly information is coupled between or among brain regions.

The data were taken from a resting-state fMRI experiment in which a subject was watching and maintaining alert wakefulness, but not performing any other behavioral task. Meanwhile, the BOLD signal was recorded. These data were downloaded from Nitime (https://nipy.org/nitime/index.html accessed on 12 October 2022). The data were preprocessed, and time series were extracted from different Regions Of Interest (ROIs) in the brain. The ROIs’ abbreviations and related full names are listed as follows: Cau, Caudate; Pau, Paudate; Thal, Thalamus; Fpol, Frontal pole; Ang, Angular gyrus; SupraM, Supramarginal gyrus; MTG, Middle Temporal Gyrus; Hip, Hippocampus; PostPHG, Posterior Parahippocamapl Gyrus; APHG, Anterior Parahippocamapl Gyrus; Amy, Amygdala; ParaCing, Paracingulate gyrus; PCC, Posterior Cingulate Cortex; Prec, Precuneus; R, Right hemisphere; L, Left hemisphere. First, we estimated the pairwise functional connectivity metrics with Pearson correlation, mutual information, and the corresponding functional connectivity, a circle-weighted graph used to visualize the outcome of pairwise functional connectivity. In Figure 6, top row (left and right), Pearson correlation and mutual information estimate the same pairwise dependencies, but later approaches capture stronger weights between ROIs, such as LPCC and RPCC, LThal and RThal, and LAmy and RAmy.

**Figure 7:**
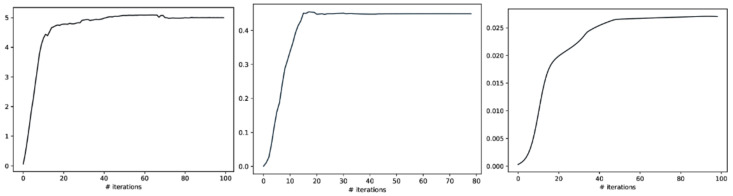
The Total Correlation convergence curve of CorEx in Layers 1, 2, and 3 is shown above. From left to right, their corresponding Layer 1, Layer2, and Layer3 parameters are selected in event-related experiments, and it shows that the Total Correlation lower bound stops increasing and tends to converge.

Meanwhile, we also used weighted graph theory to cluster dependence among ROIs, and we thresholded edges with a weight of less than 0.16 for legibility with the CorEx approach. As we mentioned above, mutual information only estimates a more robust relationship between ROIs compared to correlation. However, when we go beyond pairwise ROIs, CorEx captures richer information among all ROIs (see Figure 6 (bottom row)). Here, we selected $m_{1}=10$, $m_{2}=3$, $m_{3}=1$ as the latent dimension for each layer in our estimate of TC with CorEx, and their corresponding convergent curves are plotted in Figure 7; it shows the Total Correlation lower bound stops increasing. Figure 6 (bottom row) shows the overall structure of the learned hierarchical model. Edge thickness is determined by $\alpha_{i,j}I\left(X_{i}:Y_{j}\right)$. The size of each node is proportional to the Total Correlation that a latent factor explains about its children. The discovered structure captures several significant relationships among ROIs that are consistent with correlation and mutual information results, e.g., LPCC and RPCC, LThal and RThal, LParaCing and RParaCing, and LPut and RPut. Furthermore, TC discovered some beyond pairwise unknown relationships; for example, LCau, RCau, LFpol, and RFpol are clustered under Node 0, which explains why they have dense dependency during this cognitive task compared to other ROIs in the brain.

### 5.2. Large-Scale Connectome with Resting-State fMRI

#### 5.2.1. A Selection of Pre-Defined Atlas

**Figure 8:**
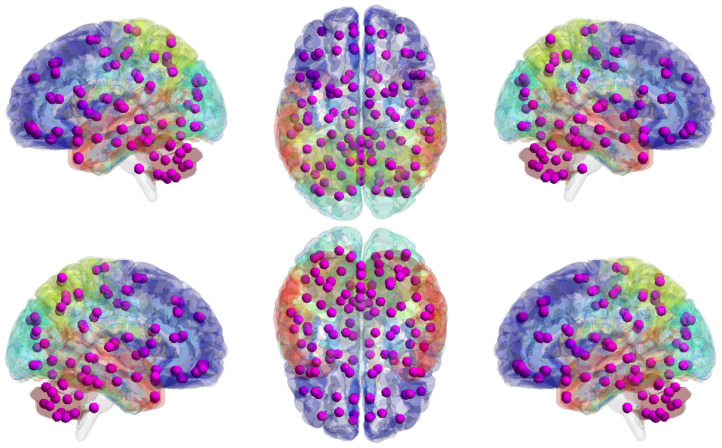
Automated Anatomical Labeling (AAL) atlas. The graph shows the volume of AAL (116 regions) mapped to the smoothed Colin27 brain surface template. The different brain areas are labeled on the brain surface with different colors, and detailed ROI/purple node information can be found in the Appendix A with Table A1.

We used the Automated Anatomical Labeling (AAL) atlas [44], a structural atlas with 116 ROIs identified from the anatomy of a reference subject (see Figure 8).

#### 5.2.2. Time Series Signals Extraction

The HCP and ACPI can access raw and preprocessed data, as well as phenotypic information about data samples. The raw rs-fMRI data were preprocessed using the Configurable Pipeline for the Analysis of Connectomes, an open-source software pipeline that allows for automated rs-fMRI data preprocessing and analysis. We extracted time series for each ROI in each subject after defining anatomical brain ROIs with the AAL atlas. We calculated the weighted average of the fMRI BOLD signals across all voxels in each region. Furthermore, the BOLD signal in each region was normalized and subsampled by the repetition time. Finally, we averaged all of the subjects’ time series signals in each ROI.

#### 5.2.3. HCP900

**Figure 9:**
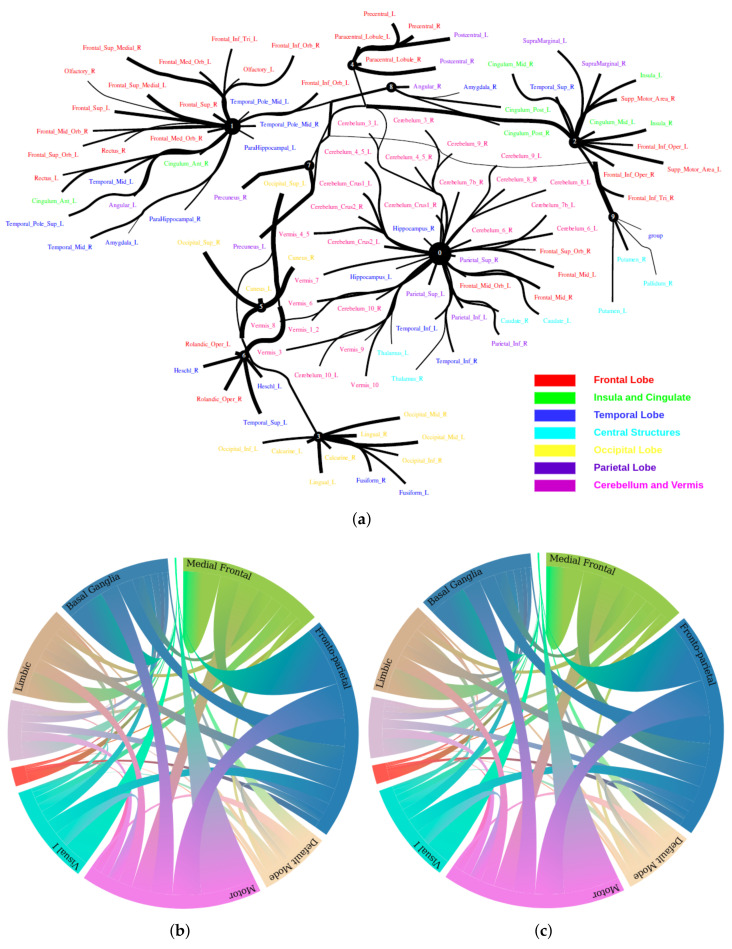
Large-scale functional connectivity with the HCP900. The functional connectivity is represented in the tree (**a**) and cycle (**b**,**c**) graphs. Top row: A weighted threshold graph with a max of 86 edges showing the overall structure of the representation learned from AAL ROIs (a high-resolution figure is represented in the appendix with Figure 10). Edge thickness is proportional to mutual information, and node size represents Total Correlation among children. In the node with red color, the frontal lobe is represented, while green color represents the insula and cingulate regions, blue color the temporal lobe, cyan color the central areas, gold color the occipital lobe, purple color the parietal lobe, and deep pink color the cerebellum and vermis. Bottom row: Two representative connectomes are presented in the form of a circular chord that shows the connections of all 116 nodes with (**b**) correlation and (**c**) mutual information of the HCP dataset. Each lobe was labeled with a different color.

The Human Connectome Project contains imaging and behavioral data from healthy people [30]. To investigate resting-state functional connectivity, we used preprocessed rest-fMRI data from the HCP900 (https://www.humanconnectome.org/ (accessed on 12 March 2021)) release [31]. Here, we selected $m_{1}=10$, $m_{2}=5$, $m_{3}=1$ as the latent dimension for each layer in our estimate of TC with CorEx. We thresholded edges with a weight of less than 0.16 for legibility. Figure 9 shows that whole-brain resting-state functional connectivity is estimated with CorEx compared to Pearson correlation and mutual information. It mostly captures relationships among brain regions, and neighboring brain regions cluster together and communicate with other areas, e.g., Node 0 has a bigger node size than other nodes.

From Figure 9, we found that brain regions are functionally clustered together, which is also consistent with structure connectivity based on their physical connectivity distance. For example, under Node 0, the cerebellum and vermis regions densely cluster together, while under Node 1, the frontal lobes cluster together and are also densely functionally connected with the temporal lobe, and so on. The different colors indicate different brain regions, which are based on Table A1. In addition, we can see that functional integration and separation exist in our brain from Figure 9.

#### 5.2.4. Computational Psychiatry Applications with ACPI

**Figure 11:**
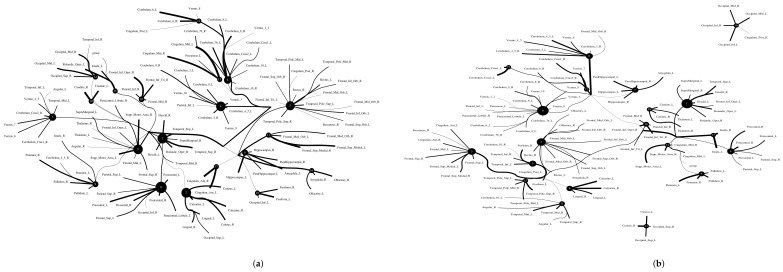
Functional connectivity between healthy group and patient group. A weighted threshold graph showing the overall structure of the representation learned from ALL ROIs. Edge thickness is proportional to mutual information, and node size represents Total Correlation among children. Here, we selected $m_{1}=20$, $m_{2}=3$, $m_{3}=1$ as the latent dimension for each layer in our estimate of TC with CorEx. (**a**) refers to normal people’s functional connectivity, and (**b**) shows the marijuana group’s functional connectivity in the brain. Both groups were measured with a TC that used the same parameters in the model. In comparison with the healthy group, we found less functional connectivity happened in the patient group, e.g., frontoparietal lobe and default model regions. (A high-resolution figure is represented in the appendix with Figure 12 and Figure 13.)

The Addiction Connectome Preprocessed Initiative is a longitudinal study to investigate the effects of cannabis use among adults with a childhood diagnosis of ADHD. In particular, we used readily preprocessed rest-fMRI data from the Multimodal Treatment Study of Attention Deficit Hyperactivity Disorder (MTA).We attempted to use functional connectivity as a bio-marker to discriminate whether individuals have consumed marijuana or not (62 in the marijuana group vs 64 in the control group). In a comparison of whole-brain functional connectivity between the control and patient groups, we found altered functional connectivity in the patient group compared to the healthy group (see Figure 11). We quantified the difference between the patient group and the healthy group, and the purity of the patient group compared to the control group was 0.85±0.23. The significant altered functional connectivity happened between the frontoparietal and motor regions. Meanwhile, we found sparse functional connectivity in the patient group compared to the control group in general. Meanwhile, we also discovered that marijuana users had more interaction between neural time series in particular ROIs such as the cerebellum, frontoparietal, and default model regions than controls, e.g., cerebellum regions mainly densely cluster around Node 0 compared to the control group. It also may explain differences in behavior in marijuana users because the frontoparietal network controls cognitive behavior execution and decision-making, cerebellum-related action, and default model network dysfunction in addicted users. All the above results are consistent with previous related research [45,46,47]. Moreover, we found some unknown disconnect between some visual regions and other brain areas. Based on related research [48,49], we suggest that marijuana patients may have altered visual perception as well.

## 6. Discussion

This manuscript presents a higher-order information-theoretic measure to estimate functional connectivity. We estimated Total Correlation with CorEx under different situations. However, the approach has its own pros and cons, which we will discuss later. Furthermore, we found that Total Correlation can be a metric to estimate functional connectivity in the human brain. It can identify some well-known functional connectivities and capture a few unknown nonlinear relationships among brain regions as well. To the best of our knowledge, this is the first time that Total Correlation has been used to estimate larger-scale functional connectivity for a whole-brain AAL atlas with 116 structural ROIs. Total Correlation can also be a tool to find biomarkers to help us diagnose brain-related diseases.

Here, we discuss some advantages and limitations of this research now. Firstly, given the curse of dimensionality of fMRI, we need to find a low-dimensional representation that helps us characterize the connectivity. Traditional General Linear Models (GLMs), such as expert-defined ROIs or the ALL atlas, are frequently used to find ROIs in resting-state experiments. However, we should be able to do better with a data-driven approach. Sample sizes and statistical thresholds are known to have a major impact on the statistical power and accuracy of GLM-based ROI selection. Previous research has revealed that the GLM has limited statistical power when inferring from fMRI data [50,51]. However, we used GLM-based ROI selection in the real fMRI datasets, which may affect the final result when we estimate functional connectivity.

Second, CorEx is model-independent, which means no anatomical or functional prior knowledge is required to estimate the ROIs. The method is entirely data-driven; this way, it is possible to analyze networks that have not been investigated and could be a future extension of work. It is also possible to use Total Correlation as a pre-analysis for other techniques such as dynamic causal modeling, which need constraints about the underlying network [52]. What differentiates the CorEx algorithm is that it tries to break the variables into clusters with high TC. In other words, CoRex finds a tree of latent factors that explain Total Correlation, so this tree of clusters based on TC is a more data-driven way to define regions and then connectivity than ROIs predefined by hand. This prioritization of “modular” solutions in CorEx was not realized or emphasized in the original research. The second reason why we used CorEx to estimate functional connectivity on larger-scale fMRI datasets is that it is a clustering approach via TC. Furthermore, CorEx estimates Total Correlation via hierarchical maximization correlation between previous layer and current layer variables with a tight information bound that estimates a more accurate relationship among variables in real neural signals.

Third, TC is an indirect information quantitative tool that cannot determine the direction of information flow between brain regions. Meanwhile, we discovered some unknown functional connectivity in the real fMRI dataset before.

Fourth, given the irregularity of neural time series and the difficulties in quantifying graph signals when brain networks are represented by graphs, we should avoid quantifying too many graph signals. However, there is a metric called permutation entropy that gives us the possibility to quantify the graph signal in complex systems [36]. It could be very interesting to apply this metric to brain networks to check how much information could be obtained from the complex graph signals, which could then help us more deeply understand brain networks in the future. Moreover, as we mentioned the complexity of neural time series, one of the important potential problems is the length of time series, except for the additional dimensional problem. It is a significant challenge when you are processing long lengths of time series, but it could be solved by transforming the time series into embedding space or segmenting the long time series into specific time windows [53].

Finally, we applied TC to estimate large-scale functional connectivity with the real fMRI dataset across the HCP and ACPI. The functional connectivity with the HCP900 gives us the potential to estimate a full brain atlas with TC in the future, and our result shows that TC can capture the right functional connectivity; beyond this, it could also give us some unknown functional connectivity. Therefore, it could be a future extension project. Furthermore, we used TC as a possible method to find biomarkers of brain disease with the ACPI dataset. We compared whole-brain functional connectivity between control and patient groups. We found altered functional connectivity in the patient group compared to the healthy group, and we quantified this difference with purity metrics because it is a simple and transparent evaluation measure. The purity in the patient group compared to the control group is not too large, and it shows that there is some altered functional connectivity in the patient group; for instance, we mentioned brain networks in the cerebellum, frontoparietal, and default model regions. However, it was just examined with one dataset with a small number of subjects and does not consider within-subject variability, and it could be extended with more large datasets in the future.

## 7. Conclusions

We introduced Total Correlation to capture multivariate large-scale interactions within brain regions. They were experimentally verified as effective steps for reconstructing multivariate relationships in the brain. In this study, CorEx was adopted to estimate Total Correlation. The CorEx approach can capture functional connectivity characteristics when going beyond pairwise brain regions. On the other hand, we evaluated the method with resting-state fMRI datasets. We found that multivariable relationships cannot be detected if we use pairwise correlation and mutual information quantities only. More generally, multivariable relationships can be clustered only if we use Total Correlation. Therefore, Total Correlation measures are significant to find complicated functional connectivity among brain regions. Furthermore, we showed that Total Correlation can estimate functional connectivity in the real neural dataset and find biomarkers for diagnosing brain diseases.

In the future, we plan to use the functional connectivity relationships discovered by Total Correlation as an input to existing Graph Neural Networks (GNNs) [54] for the purpose of interpretable brain disease diagnosis, such that practitioners or doctors can identify the most informative subgraphs (or modules) to the decision (e.g., autism patients or healthy control groups). In this regard, quantitative measures to define differences between graphs [55] and the extension of analytical results in [25] to a larger number of nodes will be critical to assess and improve the qualitative results presented here. The recently proposed approaches (e.g., [56,57]) all rely on pairwise relationships estimated by the linear correlation coefficient as the input, which ignores high-order dependence essentially. In this sense, we believe our approach has the potential to improve the explanation performances of existing GNNs on brains.

## Figures and Tables

**Figure 10:**
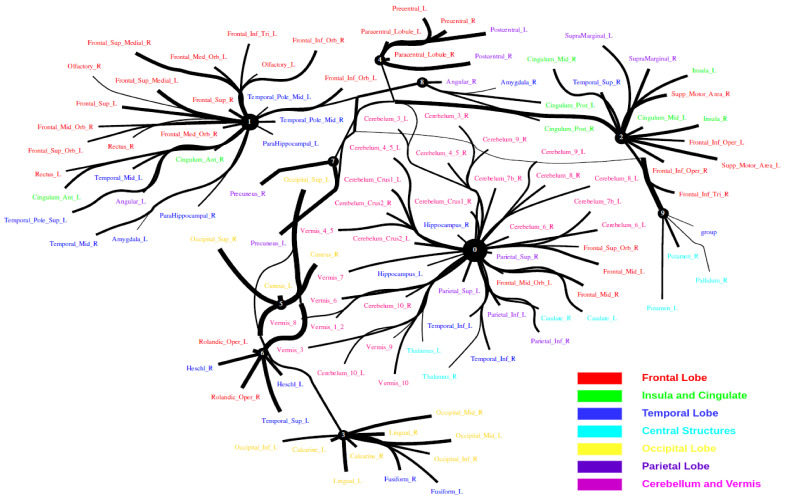
Functional connectivity of HCP900.

**Figure 12:**
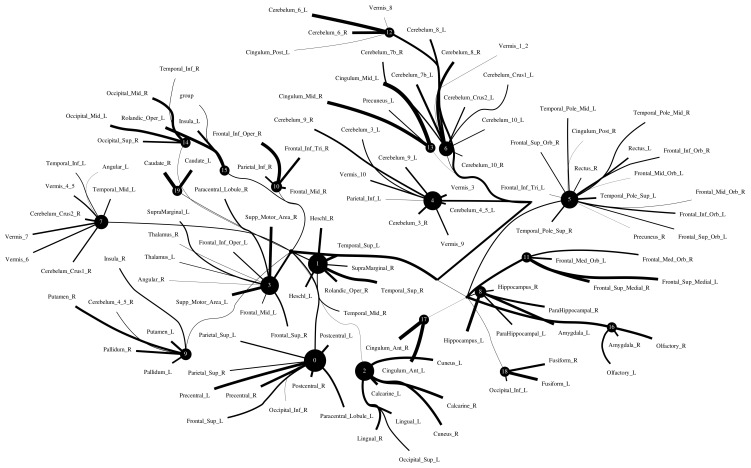
Functional connectivity of healthy group.

**Figure 13:**
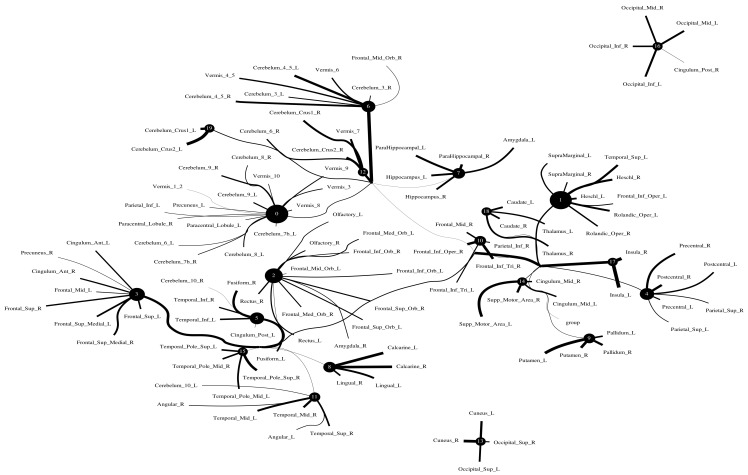
Functional connectivity of patient group.

## Data Availability

The data and code needed to reproduce the results presented here are available at https://forms.gle/1DXDpEpi7AodQ77q7 accessed on 6 November 2022.

## Abbreviations

TC	Total Correlation
CorEx	Correlation Explanation
CC	Linear Correlation
I	Mutual Information
VAEs	Variational Autoencoders
fMRI	functional Magnetic Resonance Imaging
BOLD	Blood-Oxygen-Level-Dependent Imaging
DCM	Dynamic Causal Modeling
GLM	General Linear Model
ROI	Region Of Interest
HCP	Human Connectome Project
MTA	Multimodal Treatment of Attention Deficit Hyperactivity Disorder
GNNs	Graph Neural Networks

## Appendix A

**Table A1:** Information of 116 brain regions that comprises the AAL atlas.

Brain Area	AAL Regions	AAL Index No.
	Precentral gyrus	1, 2
	Superior frontal gyrus, dorsolateral	3, 4
	Superior frontal gyrus, orbital part	5, 6
	Middle frontal gyrus	7, 8
	Middle frontal gyrus, orbital part	9, 10
	Inferior frontal gyrus, opercular part	11, 12
	Inferior frontal gyrus, triangular part	13, 14
Frontal Lobe	Inferior frontal gyrus, orbital part	15, 16
	Rolandic operculum	17, 18
	Smentary motor area	19, 20
	Olfactory cortex	21, 22
	Superior frontal gyrus, medial	23, 24
	Superior frontal gyrus, medial orbital	25, 26
	Gyrus rectus	27, 28
	Paracentral lobule	69, 70
	Insula	29, 30
Insula and	Anterior cingulate and paracingulate gyri	31, 32
Cingulate	Median cingulate and paracingulate gyri	33, 34
	Posterior cingulate gyrus	35, 36
	Hippocampus	37, 38
	Parahippocampal gyrus	39, 40
	Amygdala	41, 42
	Fusiform gyrus	55, 56
Temporal	Heschl gyrus	79, 80
Lobe	Superior temporal gyrus	81, 82
	Temporal pole: superior temporal gyrus	83, 84
	Middle temporal gyrus	85, 86
	Temporal pole: middle temporal gyrus	87, 88
	Inferior temporal gyrus	89, 90
	Caudate nucleus	71, 72
Central	Lenticular nucleus, putamen	73, 74
Structures	Lenticular nucleus, pallidum	75, 76
	Thalamus	77, 78
	Calcarine fissure and surrounding cortex	43, 44
	Cuneus	45, 46
Occipital	Lingual gyrus	47, 48
Lobe	Superior occipital gyrus	49, 50
	Middle occipital gyrus	51, 52
	Inferior occipital gyrus	53, 54
	Postcentral gyrus	57, 58
	Superior parietal gyrus	59, 60
Parietal	Inferior parietal, but supramarginal and angular gyri	61, 62
Lobe	Supramarginal gyrus	63, 64
	Angular gyrus	65, 66
	Precuneus	67, 68
	Cerebellum Crus 1	91, 92
	Cerebellum Crus 2	93, 94
	Cerebellum 3	95, 96
	Cerebellum 4, 5	97, 98
	Cerebellum 6	99, 100
	Cerebellum 7b	101, 102
	Cerebellum 8	103, 104
	Cerebellum 9	105, 106
Cerebellum and Vermis	Cerebellum 10	107, 108
	Vermis 1, 2	109
	Vermis 3	110
	Vermis 4, 5	111
	Vermis 6	112
	Vermis 7	113
	Vermis 8	114
	Vermis 9	115
	Vermis 10	116
